# Is Early Surgical Treatment for Benign Prostatic Hyperplasia Preferable to Prolonged Medical Therapy: Pros and Cons

**DOI:** 10.3390/medicina57040368

**Published:** 2021-04-09

**Authors:** Cora Fogaing, Ali Alsulihem, Lysanne Campeau, Jacques Corcos

**Affiliations:** 1 Department of Surgery/Urology, McGill University, 845 Rue Sherbrooke Ouest, Montréal, QC H3A 0G4, Canada; cora.fogaing@mail.mcgill.ca (C.F.); lysanne.campeau@mcgill.ca (L.C.); 2 Department of Urology, Prince Sultan Military Medical City, P.O. Box 7897, Riyadh 11159, Saudi Arabia; ali.alsulihem@mail.mcgill.ca

**Keywords:** benign prostatic hyperplasia, lower urinary tract symptoms, transurethral resection of the prostate, alpha-blocker, alpha-adrenoceptor antagonist, 5 alpha-reductase inhibitor, complications, green light, Holep, laser

## Abstract

*Background and objectives:* Treatment of lower urinary tract symptoms (LUTS) related to benign prostatic hyperplasia (BPH) has shifted over the last decades, with medical therapy becoming the primary treatment modality while surgery is being reserved mostly to patients who are not responding to medical treatment or presenting with complications from BPH. Here, we aim to explore the evidence supporting or not early surgical treatment of BPH as opposed to prolonged medical therapy course. *Materials and Methods:* The debate was presented with a “pro and con” structure. The “pro” side supported the early surgical management of BPH. The “con” side successively refuted the “pro” side arguments. *Results:* The “pro” side highlighted the superior efficacy and cost-effectiveness of surgery over medical treatment for BPH, as well as the possibility of worse postoperative outcomes for delayed surgical treatment. The “con” side considered that medical therapy is efficient in well selected patients and can avoid the serious risks inherent to surgical treatment of BPH including important sexual side effects. *Conclusions:* Randomized clinical trials comparing the outcomes for prolonged medical therapy versus early surgical treatment could determine which approach is more beneficial in the long-term in context of the aging population. Until then, both approaches have their advantages and patients should be involve in the treatment decision.

## 1. Introduction

Benign prostatic hyperplasia (BPH) is a slowly progressive disease affecting men typically after the fifth decade. Pathophysiology is relatively unknown limiting its prevention. BPH typically results in benign prostatic enlargement (BPE) which in turn can lead to benign prostatic obstruction (BPO), which is a symptomatic condition characterized by voiding difficulty. The treatment of lower urinary tract symptoms related to BPH has shifted over the few previous decades, medical therapy becoming the primary management in symptomatic patients. The mainly used medications are alpha-adrenoceptor antagonists (ARAs) and 5 alpha-reductase inhibitors (5ARIs). Multiple large randomized controlled studies [1,2,3] have confirmed the efficacy of medical therapy in improving LUTS symptoms caused by BPH. With the widespread use of medical treatment, the surgical procedure has been generally deferred. In most cases, only patients not responding to pharmacological management or presenting a complication are offered surgery, often years after introduction of medical therapy. We are rising concerns regarding the effect of this delay on complexity of surgeries as multiple observational studies have noticed that patients undergoing surgical procedure are becoming older with higher comorbidities and larger prostates [4], potentially exposing patients to higher risk of complications. This article will discuss the pros and cons of early surgical management.

## 2. Materials and Methods

Structure of the article: Each side (pro and con) brought three arguments each to defend their position. For each argument, starting with the ones from the “pro side,” the opinion of the two groups of authors is successively presented. A discussion and a conclusion complete the article.

## 3. Pro Side

Early surgical treatment of BPH should be performed. Three arguments support this position: surgery is far more efficacious than medical treatment, delayed surgical treatment worsen postoperative outcomes and medical therapy is not side effects free and is less cost-effective than surgery.

### 3.1. Surgery Is Far More Efficacious Than Medical Treatment

Medical therapy has not been compared to surgical treatment in randomized controlled trials. Medical therapy efficacy has been confirmed on multiple RCTs. Two important long-term RCTs, the MTOPS [1] and CombAT [2,3] trials, have only 4 years follow-up periods. In MTOPS trial, finasteride alone and the combination of doxazosin and finasteride and have resulted in a decreased need for surgery or acute urinary retention (AUR) by 69 and 64 %, and 79 and 67% respectively. In CombAT trial, the overall relative risk of reduction of BPH related surgery of AUR was 65.8% at 4 years in dutasteride treatment or combination group versus tamsulosin alone group. 5 alpha reductase (5ARIs) appears to have the main determent treatment to decrease the risk of AUR and BPH related surgery. The mean reduction of IPSS was 6.3 points and the mean increase in Q max 2.4 mL/s in Combination arm in CombAT trial. In MTOPS the improvement in IPSS was 8 points and increase in Q max was 3.7 mL/s. Unfortunately, there is no further follow up to these trials. Furthermore, those trials were conducted in a well-selected population with IPSS score less than 30 to record increase in IPSS.

In comparison, surgical treatment results in better outcomes regarding the drop of IPSS and Q max. The average increase in Q max from 25 RCT was found to be 12.7 mL/s [5]. The reported magnitude symptoms score decrease was 85% from baseline after TURP [6], which is far more prominent than medical therapy.

When looking into indirect longer-term follow-up studies, the PCPT trial follow-up showed that in patients who received finasteride or placebo, the 10-year cumulative incidence of BPH related event (LUTS, UTIs and TURP) was 36.5% in total with an absolute reduction of 6% in finasteride group which was mainly for LUTS. It was not statistically significantly different in the decrease in TURP risk [7]. Tacklind et al., have conducted a systematic review of finasteride use in BPH [8], they have found that the finasteride has an absolute risk reduction of BPH progression in 5% in 4 years, absolute risk reduction of reduction in AUR by 3% and absolute risk reduction of BPH related surgery of 3%, with longest follow up period of 4 years.

### 3.2. Delayed Surgical Treatment Worsen Postoperative Outcomes

The impact of deferred surgical therapy on detrusor function was reported indirectly in many studies. Flanigan et al. [9] found that patients who were managed initially with watchful waiting then underwent TURP had worse outcomes (Q max +4.7 and SS −8) in comparison to immediate TURP (SS −8 and Q max +8.7 mL/s). This might be due to the deleterious effect of chronic obstruction on detrusor function. Izard et al. [10] found in their series that patients presented on 1988 for TURP had a lower incidence of AUR and CUR than their patients in 2008 (22.9% and 14.6% on 1988 vs. 42.9% to 39.3% on 2008, respectively). They also found that patients discharged with catheters were higher in 2008 than in 1989 (3.2% on 1988, 12.5% on 1998 and 23.6% on 2008) along with an increase in pre-operative hydronephrosis which was lower on 1988 group (1.3% on 1988, 12.5 in 1998 and 7.1 in 2008). Similarly, Mayer et al. [11] found that patients operated on 1989 have less risk of failure to void (2.4% in 1989 to 6.2% in 2007).

The effect of medical therapy compared to surgical treatment might not have a similar impact on the bladder outlet obstruction even with improved Q max and symptoms scores. Bulut et al. [12] studied the effect of medical therapy and TURP on the bladder and prostatic vessels resistive indices. They measured the Q max, IPSS, Prostatic resistive index and bladder resistive index before the intervention and 3 months after intervention. They found that there was an improvement in all parameters except for bladder resistive index which improved after TURP but not alpha-blocker. This might explain the higher rate of retention and hydronephrosis on patients who were treated with long-term medical therapy.

Krambeck et al. have reported 13 years follow-up on patients with LUTS who underwent medical and surgical therapy in community settings [13]. They found that the patients who underwent surgical treatment were older with larger prostates and had the highest symptoms scores. Despite higher baseline characteristics, they found that only patients treated with surgical therapy (TURP or Laser vaporization) had improvement in symptoms score in comparison to the medical treatment group (5 alpha reductase inhibitors and or alpha-blockers). They found that the TURP group were the only group that had a significant reduction in incontinence after treatment.

In the group of AUR, patients treated with surgery have a better outcome than those treated with medical therapy after AUR. Lin et al. [14] have reviewed national Taiwan data for patients presented on acute urinary retention and compared the group who went into immediate TURP versus medical therapy alone. They found the TURP cohort had a lower risk of Urinary retention and UTI and decreased the risk of skeletal fractures over 3 years period.

### 3.3. Medical Therapy Is Not Side Effects Free and Is Less Cost-Effective Than Surgery

The efficacy of medical treatment is proven, especially the effectiveness of 5 alpha-reductase inhibitor in the reduction of symptoms, risk of progression, AUR and the need for surgery. Nevertheless, this medication is not free of side effects. Sexual side effects of finasteride were reported in MTOPS trial to be 4.53/100 person-years follow up for erectile dysfunction, 2.36/100 person-years follow up for decreased libido, and 1.78/100 person-years follow up for abnormal ejaculation [1]. The reported side effects of Dutasteride in CombAT trial were 7% for erectile dysfunction and 3% for decreased libido [2]. Those side effects were at 4-years period, with lack of longer follow up studies on the side effects of those medications.

Along with that, the controversial increased risk of high-grade prostate cancer which was found in the PCPT trial. There was high Gleason score (8–10) in 3.5% in finasteride group and 3.0% in the placebo group (RR 1.17, 95% CI 1.00–1.37, *p* = 0.05) [15,16]. Similarly, in the REDUCE trial, the risk of having high-grade prostate cancer in Dutasteride group was higher compared to placebo group during the third and fourth year of follow-up (12 Gleason 8–10 tumors in dutasteride group versus one in placebo group, *p* = 0.003) [17]. Those side effects and risks might be problematic for relatively younger patients who are sexually active and or having concerns about the increased risk of high-risk prostate cancer.

It is true that medical therapy has much lower rate of complications that traditional surgical approaches. However, recent advances in surgical techniques have lowered side effects related to monopolar TURP and simple prostatectomy in terms of bleeding, retrograde ejaculation and erectile dysfunction. Holmium laser enucleation of the prostate has reduced the risk of bleeding, hospitalization time and retreatment rate mainly for large prostates that would be managed traditionally with simple prostatectomy [18]. Photoselective vaporization of the prostate (greenlight laser) has eliminated the risk of TUR syndrome and significantly reduced the time of hospitalization and the risk of bleeding and blood transfusion in comparison to TURP [19]. New techniques such as water vapor therapy (rezume), prostatic urethral lift (Urolift) and water jet therapy (aquablation) have reduced the risk of sexual side effects (erectile dysfunction and retrograde ejaculation) which did not change pre- and post-operatively [20,21,22]. Water vapor therapy and prostatic urethral lift procedures have four to five-year outcome data (as long as the medical therapy RCTs) and showed better symptoms relief (IPSS scores and QOL improvement) and higher uroflow rates, along with lower sexual side effects in comparison to the medical therapy [20,21].

In regard to the costs associated with long-term combination medical therapy, Disantostefano et al. have used a Markov model over a 20-year period to evaluate the costs and effectiveness of various treatments of BPH. They demonstrated that combination therapy is more expensive than TURP after 10 years of continuous combination therapy (expected costs of TURP 8.9 (95% CI: 8.7–9.1) million dollars for TURP vs. 15 (95% CI: 12.9–16.9) million dollars for combination treatment for a cohort of 1000 men) [23]. In addition, Ahn et al. have studies their long-term cost between surgical and medical treatments for BPH. At median follow up 76 months, the mean total treatment costs of initial combination medical combination treatment were significantly higher than for patients undergoing immediate surgery (3987$ vs. 3036$, *p* < 0.001), and the out-of-pocket costs were also higher for combined medical therapy than for surgery (1842$ vs. 1436$, *p* = 0.005), both treatments were equal in costs at five years followed by increased costs of medical therapy after five years [24].

## 4. CON Side

Early surgical management of BPH should not be performed. We refute here the three arguments of the “pro” side.

### 4.1. Pro: Surgery Is Far More Efficacious Than Medical Treatment: Rebuttal; Medical Therapy Is Efficient in Well-Selected Patients; Lifestyle Changes Can Help Prevent BPH

It is undeniable that surgery can offer a quick and often durable improvement of LUTS related to BPH. However, no matter how great the efficacy of surgical treatment, up to 10% of patients may experience BPH regrowth leading to a second surgical intervention later in life [25,26] with its associated risks. Medical therapy for BPH does not offer a quick fix like surgery, but it can be sufficient to improve patient’s symptoms and related quality of life. Indeed, the MTOPS study has shown that combination of ARAs and 5ARIs significantly reduces the risk of clinical progression by 66% compared to placebo, whereas the risk reduction is more modest for each monotherapy [27]. Furthermore, the use of 5ARIs in combination with ARAs was shown to reduce the risk of AUR by 81% compared to placebo [28]. The COMBAT study, another randomized controlled trial, later confirmed that combination therapy with ARAs and 5ARIs decreased the rate of AUR and BPH-related surgery, while also decreasing significantly the IPSS score (−6.3 points at year 4) and improving the maximal flow rate (+2.4 mL/s at year 4) [2,29]. Looking more specifically at quality of life (QoL) improvement, the QUALIPROST study, a prospective, longitudinal, multicentric study carried in outpatient urology clinics, followed 1713 patients with a man IPSS score of 16.8 over a period of two years were assigned to either watchful waiting (WW), monotherapy including ARAT and 5ARIs or combined therapy (ARAT with 5ARIs). Patients treated with monotherapy and combined therapy had a significant improvement in symptoms and QoL at 6 months as measured by BII and IPSS score (−2.3 and −5.0 points respectively for treated patients) compared WW (−1.0 and −2.5 points) [30]. Furthermore, the COMBAT study reported a significantly superior improvements from baseline in terms of BII, IPSS score and patient satisfaction for combination therapy (ARAT with 5ARIs) compared to monotherapy (ARAT or 5ARIs) [31].

There is considerable amount of evidence in the literature showing that specific baseline characteristics can be used to predict the risk BPH progression. In the MTOPS placebo arm, higher PSA values and larger prostate volume measured by TRUS were associated with an increased risk of clinical progression, AUR and need for surgical intervention. Multiple studies have confirmed this association [32,33], with a general consensus based on available evidence in the literature that patients with a prostate volume > 40 mL and PSA > 1.6 ng/mL are at higher risk [34]. These characteristics should be considered when selecting patients for medical therapy as well as the duration of pharmacotherapy trial. In patients considered high risk of progression, combination of 5ARIs and ARA can still be administered to patients with features at high risk of progression as it can decrease risk of clinical progression (43.1% in the Conduct study), although by a lesser extent than for the other patients [35]. Hence, medical therapy is a treatment modality that has been proven to show great efficacy in improving LUTS and quality of life, even more so in appropriately selected patients.

Another interesting medical option that is currently emerging is the use of tadalafil, a PDE5i, in the treatment of LUTS [36]. Indeed, a recent metanalysis has shown that tadalafil 5 mg daily significantly improves LUTS as measured by reduction of IPSS score compared to placebo (SMD − 2.02 points, 95% CI = −2.52 to −1.53, *p* < 0.00001) [37]. The effect of tadalafil on LUTS is thought to be secondary to vascular and smooth muscle relaxation in the bladder neck, prostate and urethra, via the same molecular vasodilatory effect mediating its pro-erectogenic effect. Tadalafil might overall improve blood flow to the prostate and possibly prevent pelvic ischemia-related remodeling and cellular proliferation leading to BPH [37,38].

Patient education focusing on lifestyle changes will complement medical therapy as a more holistic non-invasive treatment approach to BPH. In the CONDUCT study, one third of the patients treated with watchful waiting (WW) plus lifestyle advices alone had an improvement of BPH symptoms as measured by improvement of IPSS score. A post-hoc analysis of the latter showed that WW plus lifestyle advices had similar efficacy to combination therapy of ARAs and 5ARIs in terms of IPSS score improvement, except for patients with heavy symptoms at baseline (Benign Prostatic Hyperplasia Impact Index [BII] 7–13) [39]. The lifestyle advices provided in this study were about caffeine and alcohol avoidance, bladder retraining, fluid management and avoidance of constipation [35]. Furthermore, some data suggests that a healthy lifestyle could play a role in the prevention of BPH. A prospective study involving more than 18,000 men followed from 1992 to 2008 with and without LUTS at baseline showed that men with higher BMI were more likely to develop LUTS [36]. Another study with 18,880 men demonstrated that a diet with high content of fatty food and red meat with low vegetable and protein content were associated with increased risk of BPH [40]. This dietary association was confirmed by other studies [41,42]. This data combined suggests that lifestyle modifications pertaining to balanced healthy diet and maintenance of healthy body habitus can have a beneficial effect on BPH prevention and progression. Instead of jumping to surgical management, patient education with a focus on lifestyle modifications in conjunction with medical treatment could be a valid option for patients at the early stage of BPH development and could benefit the overall health of the patient.

In conclusion, initial counselling for lifestyle changes combined with pharmacotherapy including ARAs and 5ARIs in combination or not, with adjunctive medications like tadalafil, anticholinergic or b3 agonists, doesn’t burn any bridges for eventual surgical intervention in men suffering from LUTS. This approach in selected patients will control symptoms and prevent disease progression in a less invasive manner with lower risk of irreversible complications.

### 4.2. Pro: Delayed Surgical Treatment Worsen Postoperative Outcomes; Rebuttal: Pre- and Post-Medical Therapy Era Outcomes Are Similar

The advent of medical therapy for the treatment of LUTS related to BPH in the last decades has significantly delayed surgical management, given its efficacy in managing symptoms and improving quality of life. In this context, patient undergoing TURP or other forms of surgical intervention are now overall older, with larger prostate volume and more severe comorbidities [34,43]. Despite the increasing complexity of patient presentation at the time of surgical intervention, the balancing effect of advances in surgical techniques and equipment leads to similar rates of complication. Many cohort studies looking at men undergoing TURP ranging from 1985 to 2014 have found decline over time in transfusion rate, earlier catheter removal and earlier patient’s discharge in contemporary cohorts [4,44], as well as decline in secondary hemorrhage within first postoperative 30 days and demand for re-intervention within one year [44]. There was overall no significant change in postoperative complications in more recent cohorts of patients undergoing TURP despite their older age and larger prostate volume [34]. The same observation was made in a cohort of patients undergoing laser prostate surgery, as perioperative complication rate, reoperation rate and long-term adverse effects were comparable before and after the advent of medical therapy, despite slightly longer operative time [45]. Laser technology such as HOLEP or PVP have been associated with lower blood loss compared to the traditional monopolar TURP [26]. Additionally, HOLEP now provides a minimally invasive option to treat larger prostates and has been found to have similar outcomes as open prostatectomy in terms of efficacy, but with improved safety profile including lower blood loss and shorter hospital stay [46]. Hence, current available data indicates that postoperative outcomes have not been worsened by the advent of medical therapy. Patients can now be safely treated at an older age despite having a larger prostate or comorbidities at that point, if ever medical therapy becomes insufficient to manage their symptoms.

Some fear that delaying surgical treatment of BPH can lead to permanent damage to the detrusor muscle and subsequent poor voiding abilities despite outlet obstruction relief [11]. Indeed, there is evidence that mechanosensitive epithelial and smooth muscle cells in the bladder wall, in response to sustained mechanical stretch stress caused by bladder outlet obstruction, undergo modifications of gene expression and protein synthesis ultimately leading to bladder wall remodeling and impaired detrusor contractile function [47]. This phenomenon has been observed in multiple studies supporting the increased propensity of unfavorable surgical outcomes in men who experienced acute urinary retention (AUR) preoperatively, including longer hospital stay, higher incidence of UTI within three months after surgery, increased risk of failure to void after TURP and increased rate of being discharged at home with catheter [48,49,50]. Chronic urinary retention has also been associated to poorer postoperative outcomes in terms of IPSS, Qmax and PVR [51]. However, it is important to note that the data supporting unfavorable post-operative outcomes in men with pre-operative urinary retention is derived from older studies dating from before the era of medical therapy, when the only two options were watchful waiting or TURP. Thus, these results should be interpreted in the current context with caution. Whether prolonged medical therapy delaying surgical management may cause deterioration of detrusor function remains to be elucidated. Indeed, there has not been a prospective study investigating the impact of long-term pharmacological treatment and delayed surgical management on the surgical outcomes. Patients under medical therapy are usually followed regularly by their treating urologist and would not typically be left to progress to the point of no return where irreversible ultrastructural detrusor damage resulting from prolonged CUR is present before being directed to surgical treatment. Moreover, prolonged medical therapy doesn’t seem to increase the incidence of patients progressing to AUR, as studies report that rates of AUR incidence decreased significantly (*p* < 0.001) [44] or stayed similar [4] between the pre and post-medical therapy era. This reinforces the notion that delaying surgical treatment by opting for medical therapy does not increase the likelihood of urinary retention post-operatively. Hence, the potential deleterious effects of medical management and delayed surgical management on post-operative outcomes do not apply to most patients treated medically and should not be an argument against the use of medical therapy in patients presenting with LUTS secondary to BPH.

### 4.3. Pro: Medical Therapy Is Not Side Effects Free and Is Less Cost-Effective Than Surgery; Rebuttal: Surgery Is More Risky, Higher Sexual Side Effects

The safety of medical therapy for BPH is well established as it has been thoroughly studied over the last decades and its side effects have been well described through multiple randomized controlled trials. Selective subtype alpha 1a adrenergic receptor antagonists (ARAs), namely tamsulosin and silodosin, and long-acting ARAs like alfuzosin are generally more prescribed nowadays than older nonselective ARAs such as doxazocin and terazocin. This can be explained by the fact that the latter require dose titration and can lead to serious cardiac side effects. Indeed, doxazocin has been associated to higher risks of congestive heart failure, stroke and angina when used as an anti-hypertensive in the ALLHAT study [52]. Selective ARAs, on their part, show excellent safety from a cardiovascular profile perspective and are associated with relatively benign side effects such as dizziness, rhinitis, headache and retrograde ejaculation, with silodosin having the highest rate of the latter [53]. 5ARIs, namely dutasteride and finasteride, also have an excellent safety profile and can cause sexual side effects in a minority of patients. Their main adverse effects include decreased libido (6%), decreased ejaculatory volume (3.7%), erectile dysfunction (8.1%), breast tenderness (1%) and rash (1%) [53]. Contrary to what was previously believed, recent evidence supports that 5ARIs do not increase the risk of developing high grade prostate cancer. Indeed, two recently published long-term epidemiology studies have shown that 5ARIs can reduce the overall risk of prostate cancer by up to 21% and do not increase the risk of high-grade prostate cancer [54,55]. Furthermore, an extension of the PCPT trial has shown no increase in mortality among patients using finasteride after 18 years follow up [16,56]. To summarize, medical therapy for BPH has been proven to be safe on both the short and long term and well tolerated by the vast majority of patients.

For patients who do not thrive on medical therapy secondary to side effects or insufficient efficacy, it is always possible to discontinue the offending agent and try another one, whereas side effects resulting from surgical intervention are hardly modulable and can be irreversible. Surgical intervention in itself poses multiple risks including the non-negligible risk of anesthesia, the risks of the procedure itself and additional risks pertaining to technical errors depending on the experience of the surgeon. Some patients with a risk-adverse mentality would rather try first medical therapy to potentially avoid altogether the risks of surgery. Even if performed by an experienced hand and under uncomplicated anesthesia, surgical management of BPH poses multiple risks. Monopolar TURP, the most widely used technique and the current gold standard, has been associated to the following complications [49,57]: bleeding requiring transfusion (3%), TUR syndrome (1%), urinary retention (3–9%), urinary infection (3.5%), iatrogenic incontinence (0.5%), urethral stricture (2–9%), bladder neck contracture (0.3–9%) and even mortality (0.25%). Concerning sexual side effects, TURP also displays higher risk than medical therapy. Whereas 4–26% of men treated medically with tamsulosin and up to 28% of men treated with silodosin report retrograde ejaculation [58], a much higher proportion of patients treated surgically have the same complaint, up to 65% after monopolar TURP [59]. Similarly, rates of erectile dysfunction post TURP have been reported to vary between 3.4–32% in the literature [57] as the neurovascular bundle can be damaged by transmitted electrocautery energy resulting from resection close to the prostatic capsule [60]. This compares to a more predictable rate of 8.1% of erectile dysfunction for patients treated with 5ARIs and that can be potentially reversed by the discontinuation of medication. Bipolar TURP has been shown to have an equivalent efficacy and safety profiles to monopolar TURP, with the advantage of lower rates of TUR syndrome and clot retention [61]. Concerning laser prostate surgery, a retrograde ejaculation incidence with HOLEP is around 70% [60,62], significantly higher than medical therapy, with mixed results concerning its effect on erectile dysfunction. The sexual effects following PVP remain contradictory in the literature [62].

In recent years, multiple minimally invasive techniques, including prostatic urethral lift (PUL), Rezum, aquablation, TUMT and TUNA, have been developed in an attempt to minimize the sexual side effects following surgical management of BPH. Although these novel techniques have shown lower incidence of sexual side effects compared to monopolar TURP, they often display higher rates of retreatment and are not adequate for larger prostate which limits their applicability [63]. Furthermore, their long-term safety profile and efficacy remains to elucidate given their novelty [62].

## 5. Discussion

BPH/LUTS is a condition affecting the aging man usually after the fifth decade of life and with the progressive aging of the population, it is expected to affect a growing proportion of adult men. BPH can lead to debilitating urinary symptoms, hence determining the best treatment approach is a relevant clinical question. The advent of medical therapy in the last decades with 5ARIs and ARAs has generally postponed the surgical treatment of BPH/LUTS. Pros and cons of early surgical treatment were presented above.

The main argument pro early surgical treatment of BPH/LUTS is the superior efficacy of surgery in terms of improving symptoms. Indeed, the average increase in Qmax post TURP is estimated to be 12.7 mL/s [5], while symptoms score decrease was 85% from baseline after TURP [6]. In comparison, the combination of ARAs and 5ARIs yielded a mean reduction of IPSS score of 6.3 points and a mean increase in Q max 2.4 mL/s in CombAT trial [2,3], while in MTOPS the improvement in IPSS was 8 points and increase in Q max was 3.7 mL/s [1]. Hence, although surgical treatment has never been compared head to head with medical therapy in a randomized controlled trial, current evidence points to superior results for surgery.

The pro side also argued that in addition to being more efficient, surgical treatment of BPH/LUTS is more cost-effective on the long run, with combination therapy being more expensive than TURP after five years [24] to 10 years [23] depending on the source. This indicates that the cost of prolonged medical therapy beyond 5 to 10 years could represent a higher burden on the health care system than upfront surgical treatment.

The con side explained that although not as efficient as surgery, medical therapy still offers significant improvement of BPH/LUTS and decrease the risk of clinical progression by 66% [27] and reduce the risk of AUR by 81% compared to placebo [28]. ARAs and 5ARIs have a safe side-effects profile that has been studied extensively and medical therapy avoids the risk of surgical complications including the higher rates of sexual side effects like retrograde ejaculation [59] and erectile dysfunction [57] associated with surgery. Recent data also debunks the myth that the use of 5ARIs increase the risk of high-grade prostate cancer [54,55], which reinforce the notion that medical therapy is safe on the long term. Furthermore, the con side emphasized that surgical outcomes in cohorts before and after the advent of medical therapy are comparable [34]. This seems to indicate that despite patients being older with larger prostate at the time of surgery [34,43], the complexity of the surgery can be compensated by the technical advances. Indeed, Holep now allows for surgical treatment of larger prostate size with similar outcomes as open prostatectomy in terms of efficacy, but with improved safety profile including lower blood loss and shorter hospital stay [46]. Bipolar TURP has been shown to have an equivalent efficacy and safety profiles to monopolar TURP, with the advantage of lower rates of TUR syndrome and clot retention [61]. Hence, prolonged medical therapy that delays surgery doesn’t worsen the post-surgical outcomes.

## 6. Conclusions

In conclusion, medical therapy offers the benefit of avoiding surgery with its risk of anesthesia and other complications for a large population of men with BPH. The key is to select the patients who should be given a trial of medical therapy and determine the adequate duration of treatment trial before considering surgery. Upfront surgical treatment offers better relief of LUTS secondary to BPH, but also is more risky than medical therapy including higher rates of sexual side effects. Other factors like healthcare resource management in term of cost-effectiveness and patient’s related factors like patient’s preference, desire for permanent solution and tolerability to risk should be considered in the selection of BPH treatment modality. Randomized controlled trial could clarify the effects of early surgical management versus prolonged medical therapy in BPH patients on the long-term, as those two options have never been compared in RCTs before.

## Data Availability

Not applicable.
